# Graph matching and deep neural networks based whole heart and great vessel segmentation in congenital heart disease

**DOI:** 10.1038/s41598-023-34013-1

**Published:** 2023-05-09

**Authors:** Zeyang Yao, Wen Xie, Jiawei Zhang, Haiyun Yuan, Meiping Huang, Yiyu Shi, Xiaowei Xu, Jian Zhuang

**Affiliations:** 1 School of Medicine, South China University of Technology, Guangzhou, 510006 China; 2 Guangdong Provincial Key Laboratory of South China Structural Heart Disease, Guangdong Cardiovascular Institute, Guangdong Provincial People’s Hospital (Guangdong Academy of Medical Sciences), Southern Medical University, Guangzhou, 510080 China; 3 School of Computer Science, Fudan University, Shanghai, 200433 China; 4 Department of Computer Science and Engineering, University of Notre Dame, Notre Dame, IN 46556 USA

**Keywords:** Cardiovascular diseases, Computational models, Machine learning, Paediatric research

## Abstract

Congenital heart disease (CHD) is one of the leading causes of mortality among birth defects, and due to significant variations in the whole heart and great vessel, automatic CHD segmentation using CT images has been always under-researched. Even though some segmentation algorithms have been developed in the literature, none perform very well under the complex structure of CHD. To deal with the challenges, we take advantage of deep learning in processing regular structures and graph algorithms in dealing with large variations and propose a framework combining both the whole heart and great vessel segmentation in complex CHD. We benefit from deep learning in segmenting the four chambers and myocardium based on the blood pool, and then we extract the connection information and apply graph matching to determine the categories of all the vessels. Experimental results on 68 3D CT images covering 14 types of CHD illustrate our framework can increase the Dice score by 12% on average compared with the state-of-the-art whole heart and great vessel segmentation method in normal anatomy. We further introduce two cardiovascular imaging specialists to evaluate our results in the standard of the Van Praagh classification system, and achieves well performance in clinical evaluation. All these results may pave the way for the clinical use of our method in the incoming future.

## Introduction

Congenital heart diseases (CHD) are defects in the structure of the heart or great vessels that present at birth^1^. The structure defects can involve the walls of the heart, the valves of the heart, and the arteries and veins near the heart. Each of these significant structure variations can disrupt the normal flow of blood through the heart, which cause serious health problems or death.

Clinical decision-making and surgery planning are the main challenges in the treatment of CHD. Currently, 2D (e.g., 2D echocardiography) and 3D (e.g., 3D echocardiography, magnetic resonance imaging (MRI), and computed tomography (CT)) imaging techniques are widely used to tackle these challenges. Furthermore, if surgery is required, CT and MRI are usually mandatory to help surgeons to have an informative knowledge of the heart structure. However, such a process is time-consuming and non-intuitive which introduces many difficulties to especially less experienced surgeons.

**Figure 1:**
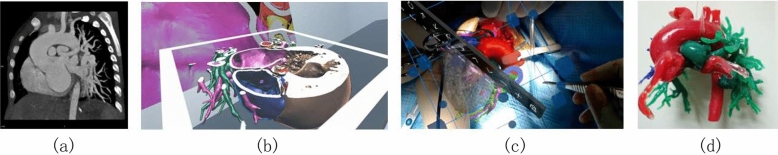
Illustration of 3D virtualization and 3D printing in CHD surgery: (**a**) is a typical coronal CT scanning of CHD; (**b**) is a horizontal section of heart segmentation results using virtualization reality (VR) for CHD surgery planning; (**c**) is a view in augmented reality (AR) for guiding real-time surgery; (**d**) is a printed model using 3D printing.

In recent years, 3D virtualization techniques (e.g., virtual reality (VR) and augmented reality (AR)) and 3D printing have been widely adopted for clinical decision-making and surgery planning of CHD^2–5^, greatly making up for the limitations of the traditional examination methods. As shown in Fig. 1, 3D medical images (usually CT or MRI) of hearts are usually used to produce a digital model of the heart which is then used for VR, AR, and 3D printing. Such virtualization of the heart can help surgeons have an intuitive sense of the structure and connections of the heart. To obtain the digital models, whole heart and great vessel segmentation are usually performed manually which is a time-consuming and costly process.

Nowadays, the development of deep learning has shown its advantages in the performance of segmentation^6,7^. There are some works in this area trying to solve the above problems^8–11^, and most of these works are based on MRI data and focus on chambers and the whole heart only^12–15^. For congenital heart disease, Mukhopadhyay et al.^16^ used total variation random forest to perform fully automatic congenital heart disease in magnetic resonance images (MRI) sequences. Li et al.^11^ proposed a deep learning method for automatic whole-heart segmentation in cardiac magnetic resonance (CMR) images with CHD. The state-of-the-art segmentation performance in computed tomography (CT) images is obtained by Payer et al.^8^, they combine 3D U-Net for segmentation and a simple convolutional neural network for label position prediction. Recently there are also some works about blood pool segmentation of CHD images^17,18^, which only handles the blood pool and myocardium.

Moreover, Pace et al.^19^ adopted a semi-automated approach for left ventricle (LV) and aorta (Ao) segmentation in CHD, they use a recurrent neural network recursively evolves segmentation in several steps, which requires user interaction to locate an initial seed for segmentation. Yoshida et al.^20^ using U-net for investigating the usefulness of deep learning methods for segmenting the whole heart region and the cardiac cavity region in pediatric cardiac CT images. However their results are achieved on a small dataset and only the Dice similarity coefficient (DSC) is used for evaluating the segmentation accuracy, which is not suitable for CHD diagnosis to some extent. Pace et al.^21^ proposed an iterative segmentation model and show that it can be accurately learned from a small MRI dataset, the model they proposed evolves a segmentation over multiple steps and achieved great performance on CHD segmentation. However, the characteristics of MRI images they used are completely different from those of CTA. In developing countries, the accessibility of CTA is far greater than that of MRI, and the study of the application of CTA in congenital heart disease is equally significant. All in all, fully automated segmentation of the whole heart and great vessel segmentation of CHD using CTA images is still an under-researched piece in the literature.

In this paper, we propose an algorithm that combines deep neural networks and graph matching for the whole heart and great vessel segmentation in CHD based on our previous work^22^. Note that graph matching has been applied in a variety of applications^23–25^, but not yet to congenital heart disease segmentation.

Particularly, we first use deep neural networks to segment four chambers and cardiac muscle, where variations are usually small. Then We extract the connection information and apply graph matching to determine the category of all the vessels. For making up for the shortcomings of Dice scores in evaluation segmentation results, two cardiovascular imaging specialists engaged to evaluate our results. Experiment results show that our method performs better than the state-of-the-art method for multi-modality whole heart and great vessel segmentation on both segmentation criterion and clinical evaluation. And as far as we know, this is the first work to perform the whole heart and great vessel segmentation and reconstruction in CHD. We hope all these contributions may pave the way to the clinical use of our method in the incoming future.

## Materials and methods

### Patient demographics

The ages of the associated patients range from 1 month to 21 years, and the average age at repair is 79.75 ± 59.07 weeks, with the majority between 1 month and 2 years. There are 40 females in the patients, approximately 60% of the total. Based on the degree of morphological variability of CHD^26^, we have divided our dataset into two categories: simple and complex. And there are 14 types of CHD, six simple types and eight complex ones, which all have been discussed in Table 1. Specifically the eight complex ones including Tetralogy of Fallot (TOF), transposition of great arteries (TGA), pulmonary artery sling (PAS), Anomalous Pulmonary Venous Connection (APVC), common arterial trunk (CAT), aortic arch anomalies (AAH), single ventricle (SV), pulmonary atresia ($PA_{atresia}$)). The number of images associated with each is summarized in Table 2.

**Table 1:** Types of CHD and their descriptions.

Types	Description
ASD	A hole in the wall (septum) that divides the upper chambers (atria) of the heart
VSD	A hole in the wall (septum) that separates the two lower chambers (ventricles) of the heart
AVSD	A large hole in center of the heart affecting all four chambers
PDA	The ductus arteriosus fails to close after birth
CoA	A part of the aorta, the tube that carries oxygen-rich blood to the body, is narrower than usual
PS	A stenosis of main pulmonary artery and/or its branches
TOF	A heart birth defect that includes 4 defects: ventricular septal defect, right ventricle outflow tract stenosis, aorta overriding and secondary right ventricular hypertrophy
TGA	The main pulmonary artery and the aorta are switched in position, or “transposed”
PAS	Left pulmonary artery originates from the right pulmonary artery, encircles the right main-stem bronchus, and distal trachea before entering the hilum of the left lung
APVC	The lungs pulmonary veins don’t connect completely or partly to the left atrium like usual
CAT	A single common blood vessel comes out of the heart, instead of the main pulmonary artery and aorta
IAA	Aorta is not completely developed, part of the aorta is missing, leaving a gap
SV	One lower chamber (ventricle) does not develop, and the heart has only one pumping chamber
$PA_{atresia}$	The valve that controls blood flow from the heart to the lungs doesn’t form at all

**Table 2:** The types of CHD in our dataset and the case number associated with them.

Simple CHD 37						Complex CHD 31								Normal
ASD	AVSD	VSD	PDA	CoA	PS	TOF	TGA	PAS	APVC	CAT	AAH	SV	$PA_{atresia}$	
17	4	26	7	4	4	7	4	3	20	4	8	2	7	2

Note that some images may correspond to more than one type of CHD.

### Image and acquisition parameters

Our dataset consists of 68 3D CTA images, and all images were obtained by a SOMATOM Definition Flash Dual-source CT scanner, using the following protocol: collimation, (96–128) $\times$ 0.625 mm; rotation time, 270 ms, which corresponds to a 135 ms standard temporal resolution; slice thickness, 0.9 mm; reconstruction interval, 0.45 mm. Adaptive axial z-collimation was used to optimize the cranio-caudal length. Data were obtained at 40–50% of the RR interval, utilizing a 5% phase tolerance around the 45% phase. The dosage is 100 kVp/651 mAs (routine-dose). The size of the images is 512 $\times$ 512 $\times$ (130–340), and the typical voxel size is 0.25 $\times$ 0.25 $\times$ 0.5 mm$^3$.

### Ground-truth labels generation

All labeling was performed by experienced radiologists, and the time for labeling each image was 1–1.5 h. Within the range from the superior plane of the clavicle to the plane of the aortic hiatus of the diaphragm, we identified seven target cardiovascular structures: the left ventricle (LV), right ventricle (RV), left atrium (LA), right atrium (RA), myocardium (Myo), aorta (Ao), and pulmonary artery (PA). During the procedure, the CTA images were imported into the Mimics software and labeled using thresholding. We assumed that the target and background of the image occupy different ranges of gray levels, with small differences in the gray values between adjacent pixels within the target and background. However, the pixels on both sides of the target-background interface have a large difference in gray value. Based on the prior knowledge of the anatomy recognized by the radiologist, we selected seven different gray threshold values to mark the anatomical structures. Then, we compared the gray values of each pixel in the image with these threshold values, and according to the comparison results, the pixels were classified into different categories. This process produced a continuous gray threshold image, and the target was extracted from the background. Next, we used the differences in characteristics such as gray level, color, and texture between the target and background to achieve cardiovascular edge detection. We first performed threshold segmentation and then mask editing. After these preprocessing steps, we used a three-dimensional reconstruction algorithm combining shear deformation to obtain the three-dimensional reconstruction of the cardiovascular model at different levels. Due to the large variability of the cardiovascular structures in congenital heart disease, vena cavae (VC) were also labeled as part of RA, and pulmonary veins (PV) were labeled as part of LA for ease of processing, as they are connected, and their boundaries are relatively hard to define. Anomalous vessels such as the delayed joining of the subclavian vein to the superior vena cava and abnormally enlarged pulmonary veins were also identified and classified as one of the seven aforementioned cardiovascular substructures based on their connections.

### Framework overview

**Figure 2:**
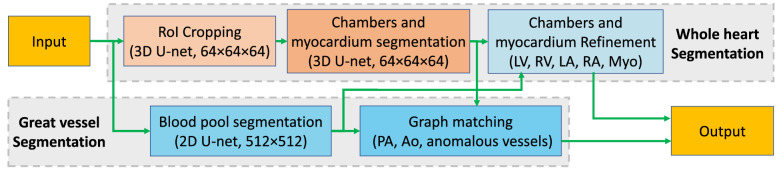
Overview of the proposed framework combining deep learning and graph matching for whole heart and great vessel segmentation in CHD.

The whole heart and great vessel segmentation method have two sub-tasks: heart segmentation and great vessel segmentation. The overall framework is shown in Fig. 2

.

### Whole heart segmentation

**Figure 3:**
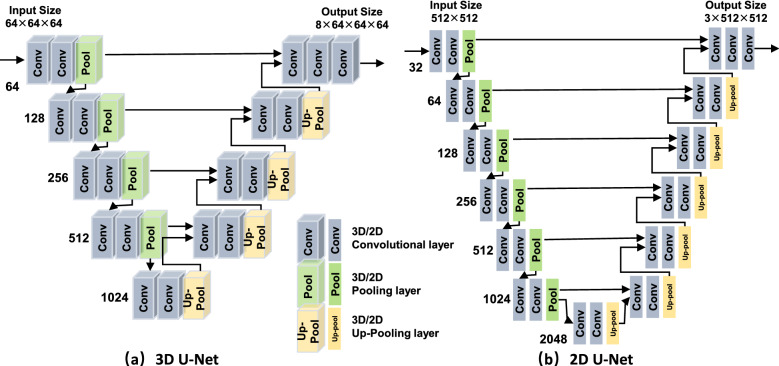
Network structures of the adopted 3D U-net and 2D U-net.

**Figure 4:**
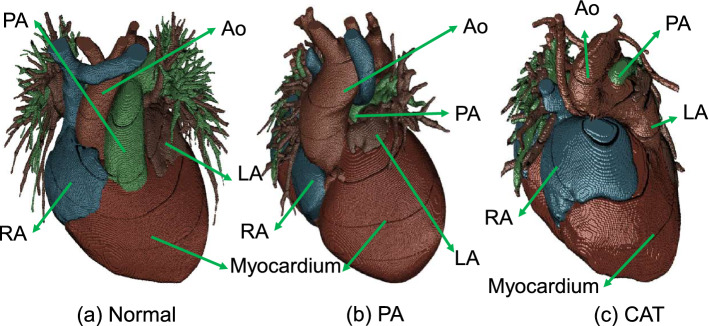
Pulmonary atresia ($PA_{atresia}$) and common arterial trunk examples (CAT) in our dataset, with large variations from normal heart anatomy.

**Figure 5:**
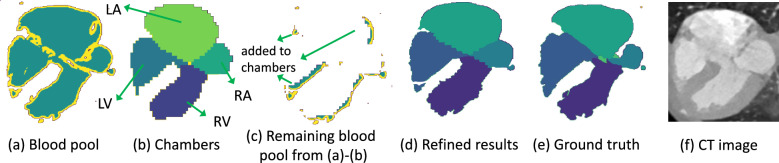
Illustration of chambers and myocardium refinement. (**a**) Is obtained from blood pool segmentation (high resolution). (**b**) Is from chambers and myocardium segmentation (low resolution). (**c**) Is the remaining blood pool by subtracting chambers (**b**) from blood pool (**a**). It is added to the surrounding chambers to refine the boundaries (**d**). (**e,f**) Are the ground truth and CT image, respectively.

Segmentation was performed with multiple U-Nets^27^, and the network structure of the adopted 3D U-net and 2D U-net is shown in Fig. 3. There were several steps in segmentation: *Region of interest (RoI) cropping* extracts the area that includes the heart and its surrounding vessels. We resized the input image to a low resolution of 64 $\times$ 64 $\times$ 64, and then adopt the same segmentation-based extraction as^8^ to get the RoI. *Chambers and myocardium segmentation* resized the extracted RoI to 64 $\times$ 64 $\times$ 64 which had fed to a 3D U-net for segmentation. *Chambers and myocardium refinement* refined the boundaries of chambers and myocardium based on the outputs of chambers and myocardium segmentation and blood pool segmentation. Due to the limited GPU memory^8^, the input of 3D U-net was usually limited to low resolution or small size, and accordingly, the chambers and myocardium segmentation results may lose boundary information. This was critical for CHD where significant variations exist. To address this issue, we refined the boundary of chambers and myocardium by reusing the blood pool segmentation results, which were in high resolution. Specifically, we removed the portion of the blood pool that corresponded to the chambers from the results of blood pool segmentation, and the remaining blood pool was added to its surrounding chambers to refine the boundaries. With the refined boundary of chambers, the boundary of the myocardium was also refined as the chambers and the myocardium share a large portion of boundaries as shown in Fig. 4. An illustration of the refinement process is shown in Fig. 5. Comparing (b) with (e), we can notice that part of the boundary information is lost, and the boundary is indeed refined after the process as shown in (d).

### Great vessel segmentation

**Figure 6:**
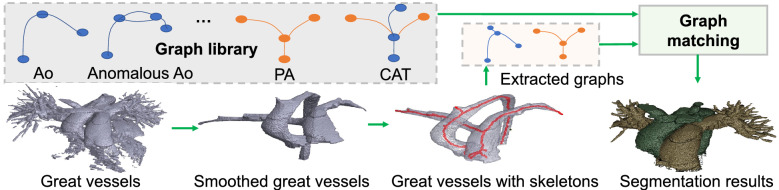
Illustration of great vessel segmentation with graph matching. With smoothing, the skeleton of great vessels can be easily extracted, and then its corresponding graph is obtained for graph-matching-based classification of Ao, PA, and anomalous vessels.

First, *blood pool segmentation* was conducted on each 2D slice of the input using a 2D U-net^27^ with an input size of 512 $\times$ 512. Note that to detect the blood pool boundary for easy graph extraction in graph matching later, we added another class blood pool boundary in the segmentation. Second, *graph matching* identified Ao, PA, and anomalous vessels using the outputs of blood pool segmentation and chambers and myocardium segmentation. Great vessels could be obtained by removing the chamber areas from the blood pool, which need to be segmented to identify Ao, PA as well as anomalous vessels. To address significant variations that occurred in CHD, we adopt a surface thinning algorithm^28^ to obtain skeletons of blood vessels for graph matching, and the workflow is shown in Fig. 6. A graph library is built based on medical knowledge to represent all the possible connections between great vessels and anomalous vessels. First, we illustrate all the possible combinations of PA and Ao, and the corresponding key points and their categories (Ao and PA) are labeled. We then extracted the graphs corresponding to Ao, PA, and anomalous vessels or their mixtures.

However, due to inaccurate blood pool segmentation or small anomalous connections, sometimes only one large graph corresponds to Ao and PA, which makes the matching difficult. Thus, these extracted graphs (corresponding to Ao and PA) should be disconnected from each other, and Ao and PA have their own corresponding graphs which can be matched with the ones in the library. To tackle this issue, we applied multiple smoothing in various scales (e.g., using a 3 $\times$ 3 $\times$ 3 kernels (all 1) 1 to 7 times iteratively for smoothing) to extract several candidate graphs. Then we matched these graphs with the ones in the library to identify the most similar pairs.

Particularly, we model the graphs as distributions, and such graph similarity is calculated using the earth mover’s distance (EMD) which is a widely used similarity metric for distributions^29^. Two factors need to be modeled: the *weight* of each bin in the distribution, and the *distance* between bins. We model each sampled point in the sampled skeleton as a bin, the Euclidean distance between the points as the distance between bins, and the volume of blood pool around the sampled point as the weight of its corresponding bin. Particularly, the weight is defined as $r^3$ where *r* is the radius of the inscribed sphere in the blood pool centered at the sampled point. With graph matching, the categories of the extracted graphs as well as the categories of the corresponding vessels in these graphs could be determined (based on the labeled graphs in the library). The vessels that left out in the smoothing process were finally classified by a simple region growing technique^30^.

### Van Praagh classification system

**Figure 7:**
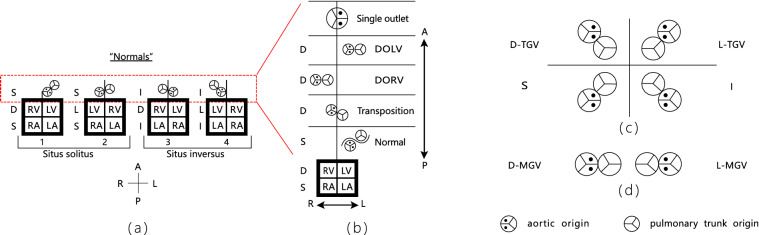
Illustration of clinical evaluation in the standard of Van Praagh classification system. (**a**) Is a model of four so-called normal hearts-that is, hearts with atrioventricular and ventriculoarterial concordant connections. The vertical line above the box represents the position of the ventricular septum. Note that in situs inversus, the aortic origin lies to the left of pulmonary trunk origin. The red dotted line represents the location of ventriculoarterial connections. (**b**) Is a model of varieties of ventriculoarterial connection. Aortic origin in the transposition of the great arteries and the double outlet ventricles (DORV, DOLV) is indicated by D when it lies to the right of the pulmonary artery origin and L when it lies to the left. (**c**) Schematically show the great vessel configurations on axial cross-sectional images at the level of valves when in a normal position (left column) or inversion (right column) and in normal relation (bottom row) or transposition (top row). (**d**) Schematically show d-malposition (top) and l-malposition (bottom) of the great vessels.

A brief illustration of the Van Praagh classification system is shown in Fig. 7. In the Van Praagh system, a three-part notation consisting of letters separated by commas and encompassed by a set of braces is used to succinctly describe the visceroatrial situs, the orientation of the ventricular loop, and the position and relation of the great vessels. Van Praagh’s symbolic representation may be combined with those of atrioventricular and ventricular arterial connections as shown in Fig. 7a–d. In Van Praagh’s convention, the first letter (S or I) refers to the atrial position (solitus or inversus), the second letter (D or L) to the ventricular loop, and the third letter to the position of the origin of the aorta (recognized by its two coronary ostia) in relation to the origin of the pulmonary trunk. The arrangement of boxes and abbreviations is identical in all similar models presented in Fig. 7.

### Experiments

All the experiments were run on an Nvidia GTX 1080Ti GPU with 11 GB memory. We implemented our 3D U-net using Pytorch based on^8^. For 2D U-net, most configurations remain the same as those of the 3D U-net except that 2D U-net adopts 5 levels and the number of filters in the initial level was 16. Both Dice loss and cross-entropy loss were used, and the loss function is as follows:

1 $$L = \left( {1 - \frac{1}{C}\sum\limits_{{j = 1}}^{C} {\frac{{2\sum\limits_{{i = 1}}^{N} {p_{i}^{j} } g_{i}^{j} }}{{\sum\limits_{{i = 1}}^{N} {(p_{i}^{j} + g_{i}^{j} )} }}} } \right) + \frac{1}{{NC}}\sum\limits_{{j = 1}}^{C} {\sum\limits_{{i = 1}}^{N} { - g_{i}^{j} \log (p_{i}^{j} )} } ,$$

where $p_{i}^{j}$ and $g_{i}^{j}$ are the predicted and ground truth label maps at pixel *i* for the *j*th class. and the training epochs were 6 and 480 for 2D U-net and 3D U-net, respectively. Data augmentation was also adopted with the same configuration as in Ref.^8^ for 3D U-net. Data normalization was the same as^8^. The learning rate was 0.0002 for the first 50% epochs, and then 0.00002 afterward.

Due to the scarcity of related CT CHD segmentation algorithms, our work is one of the pioneers in exploring and addressing this challenging task, we adopted Seg-CNN^8^ that achieves the state-of-the-art performance in whole heart and great vessel segmentation of hearts in normal anatomy for comparison. The configuration was the same as that in Ref.^8^.

For both methods, fourfold cross-validation was performed (17 images for testing and 51 images for training). The split of our dataset considers the structures of CHD so that any structure in the testing dataset also had a similar presence in the training dataset, though they may be not of the same type of CHD.

**Table 3:** Mean and standard deviation of Dice score, and paired t-test of between the state-of-the-art method Seg-CNN^8^ and our method (in %) for seven substructures of the whole heart and great vessel segmentation.

Method	LV	RV	LA	RA	Myo	Ao	PA	Overall
Seg-CNN	67.3 ± 13.9	65.0 ± 12.0	70.2 ± 7.8	76.0 ± 7.5	71.5 ± 8.3	63.0 ± 13.3	52.3 ± 12.3	66.5 ± 10.7
Our method	**82.4** ± **10.5**	**77.6** ± **14.3**	**78.6** ± **7.4**	**82.7** ± **7.5**	**77.3** ± **8.3**	**82.2** ± **8.1**	**67.1** ± **19.8**	**78.3** ± **10.8**
t-value	7.15	5.57	6.44	5.21	4.07	10.17	5.24	6.40
P	$\mathbf{<}$ **0.05**	$\mathbf{<}$ **0.05**	$\mathbf{<}$ **0.05**	$\mathbf{<}$ **0.05**	$\mathbf{<}$ **0.05**	$\mathbf{<}$ **0.05**	$\mathbf{<}$ **0.05**	$\mathbf{<}$ **0.05**

Significant values are in bold.

The Dice score was used for segmentation evaluation. For evaluation, we adopt both general segmentation criteria and clinical evaluation for the assessment of our method. The Dice score was used as a general segmentation criterion for fourfold cross-validation. For clinical evaluation, two cardiovascular imaging specialists were introduced to evaluate our segmentation results in the standard of Van Praagh classification system^31–33^.

### Ethics declarations

All data analyzed were collected as part of routine examination and diagnosis, and this data collection involves no procedures for which written consent is normally required outside the research context. All analysis procedures performed in studies involving human participants’ data were in accordance with the ethical standards of the institutional and/or national research committee and with the 1964 Declaration of Helsinki and its later amendments or comparable ethical standards. And the informed consent is waived under the approval of the Research Ethics Committee of Guangdong General Hospital, Guangdong Academy of Medical Science under Protocol No. 20140316.

## Discussion

### General segmentation results

**Figure 8:**
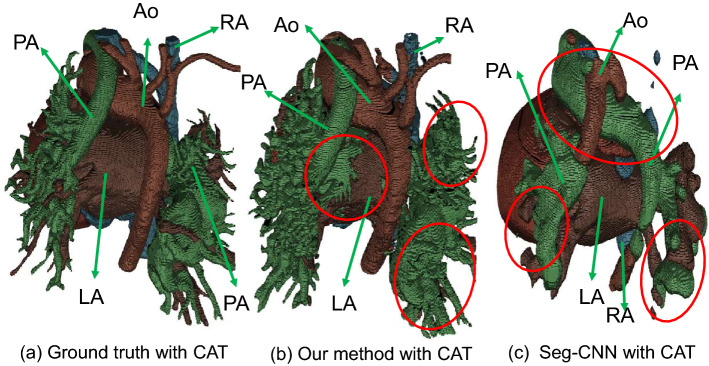
Visualized comparison between the state-of-the-art method Seg-CNN^8^ and our method. The differences from the ground truth are highlighted by the red circles.

The comparison with Seg-CNN^8^ is shown in Table 3. Our method achieves a 5.8–19.2% higher mean Dice score across all the seven substructures (12% higher on average). The highest improvement is attained in Ao, which is due to its simple graph connection with successful graph matching. The least improvement is obtained in the myocardium, which is because the myocardium is not well considered in the high-resolution blood pool segmentation. Visualization of CAT segmentation using our method and Seg-CNN is shown in Fig. 8. Our method is capable of accurately segmenting the Ao and PA, with only minor instances of incorrect segmentation between the PA and LA. However, Seg-CNN wrongly segments the main part of the Ao as PA due to the limitations of pixel-level segmentation, in which the U-net-based framework only takes into account the surrounding pixels and fails to adequately incorporate connection information.

**Table 4:** Mean and standard deviation of Dice score, and paired t-test of between the state-of-the-art method Seg-CNN^8^ and our method (in %) in simple and complex CHDs.

Types	Seg-CNN	Our method	t-value	P
Simple CHD	70.3 ± 8.3	**82.6** ± **6.2**	6.2	$\mathbf{<}$ **0.05**
Complex CHD	62.7 ± 14.4	**74.1** ± **14.5**	14.5	$\mathbf{<}$ **0.05**

Significant values are in bold.

The segmentation performances of Seg-CNN^8^ and our method in different scenarios (simple and complex CHD)^26^ are shown in Table 4. Both methods achieve higher mean Dice with lower standard deviations in simple CHD than in complex CHD, as complex CHD has more complicated structure variations. Compared with Seg-CNN, our method achieves about 12% higher average mean Dice score on both simple and complex CHD. Our method also achieves a 1.9% reduction on the standard deviation of Dice score in simple CHD compared with Seg-CNN^8^.

**Figure 9:**
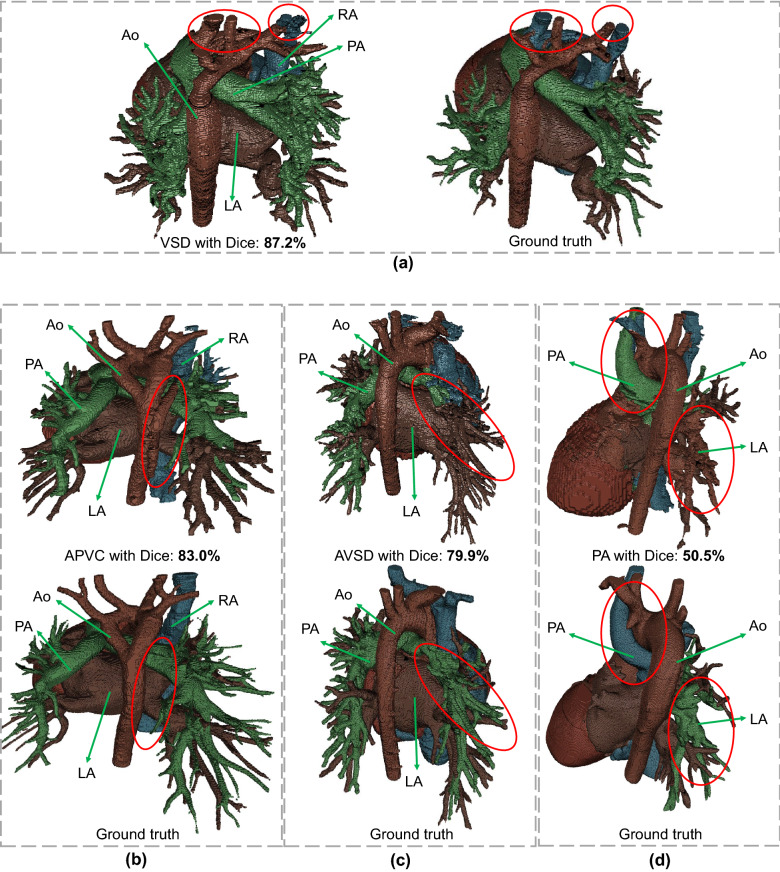
Visualization of our segmentation results with (**a**) best, (**b,c**) median, and (**d**) worst Dice score among all the test images. Their corresponding ground truth is also shown, and red circles indicate the segmentation error.

Finally, visualizations of segmentation results from our method with the best, median, and worst Dice scores among all the test images are shown in Fig. 9. The segmentation results in Fig. 9a achieve the best accuracy, and most of the structures are segmented correctly, with some errors in the connections between RA and Ao, and the wrong connections are indicated by the red circle. The segmentation results in Fig. 9b,c have some severe wrong segmentations: the one in Fig. 9b has an anomalous vein from RA which is segmented as part of Ao due to the boundary extraction error in blood pool segmentation, and the one in Fig. 9c suffers from the boundary extraction error between LA and PA. This type of error also results in the worst Dice score as shown in Fig. 9d, which corresponds to ground truth provided in Fig. 9d. In the ground truth, a thick anomalous vein from RA crosses Ao, and PA has no trunk vessels and is of a very small volume. Compared with the ground truth, the thick anomalous vein from RA is misclassified as PA, and the majority of PA is misclassified as LA.

### Clinical evaluation results

The detail of clinical evaluation results are shown in Table 5. All results were assessed independently by two cardiovascular imaging specialists, and discordant reads were reviewed and discussed until a consensus was reached. Our method had not made mistakes in some simple CHDs like ASD, AVSD, VSD, PS, and CoA. Specifically, we found the position of the atrium of a PDA case changed from situs solitus to situs inversus. More common wrong segmentations happened in complex CHD. In a TOF case, we found the ventricular loop changed from curved rightward to curved leftward. Interestingly we found the position of the aortic origin changed with pulmonary trunk origin, the aorta was anterior to and leftward of the pulmonary trunk origin, the anomaly is described as levotransposition (l-transposition) or congenitally corrected transposition, which is shown as L-TGV in Fig. 5. In a single ventricle case, we found the ventricle was segmented into the left and right two parts. The overall percentage of correct connections in atrioventricular, ventriculoarterial, and the relationship of the great vessels is 0.986 (70/71), 0.97 (69/71), and 0.986 (70/71).

**Table 5:** Results of clinical evaluation.

Types	Total cases	Wrong cases	Description
ASD	17	0	
AVSD	4	0	
VSD	26	0	
PDA	7	1	{***S*** $\rightarrow$ ***I***, D, S}
CoA	4	0	
PS	4	0	
TOF	7	1	{S, ***D*** $\rightarrow$ ***L***, S}
TGA	4	1	{S, D, ***S*** $\rightarrow$ ***L-TGV***}
PAS	3	0	
APVC	20	0	
CAT	4	0	
AAH	8	0	
SV	2	1	{S, ***X*** $\rightarrow$ ***D***, S}
$PA_{atresia}$	7	0	
Normal	2	0	

$\textit{Total cases}$ gives all case number of each type CHD, $\textit{Wrong cases}$ shows wrong segmentation results number, and $\textit{Description}$ presents the specific type of the wrong segmentation, with bold and black italics indicating specific wrong parts. Divided by types according to Table 2.

Our method achieves much better performance than the state-of-the-art segmentation method. However, in the 3D segmentation process, although each type of CHD shown in Fig. 9 achieves good accuracy under the general segmentation criterion, there are still some tiny wrong-connected segmentations. Thus far, we have considered connection features in the blood pool and considered the shapes of the vessels to improve our segmentation results. However, the presence of certain defects may be attributed to the failure of boundary extraction in blood pool segmentation. From a data perspective, the initial threshold-based construction of ground-truth labels also limits the differentiation of boundary and boundary extraction, which is also a limitation of our study and may contribute to the occurrence of these errors in segmentation. In order to enhance performance, additional structural features should be taken into account, such as local tissue changes. This requires innovative approaches from the deep learning community and deeper collaboration between computer scientists and radiologists.

Unless we can achieve excellent performance in every segmentation result, using the Dice score to evaluate our method is not a satisfactory choice. Due to the uncertainty and incomprehensibility of the U-Nets network, pixel-level segmentation criterion may result in some segmentation parts being consistent with the ground truth, while some critical parts are not. It is easy to understand from Fig. 5. In chambers and myocardium refinement, either boundaries from high-resolution segmentation results or low-resolution segment wrong will cause refined boundaries do not consistent with the ground truth, and introduce connection mutations to segmentation results. When it comes to 3D printing and virtual surgery planning, the impact of those connection mutations is serious. The existence of some basic anatomical errors is not acceptable clinically, it is necessary to introduce another criterion clinically to guarantee there is not an atrium defect from segmentation compared with the ground truth.

However, there are still no recognized clinical criteria to instruct the training process of the segmentation network. In the standard of Van Praagh classification system, our two cardiovascular imaging specialists introduced a new clinical evaluation to assess our method. Because the Van Praagh notation imposes on its users a systematic approach to anatomic description, it is a helpful device for structuring the interpretation of segmentation results as well as the reporting of evaluation. By manual evaluation of the segmentation results of each case, we roughly proved the clinical availability of our method. From detailed check results from Table 5, we can notice that most types of CHD achieve correct segmentation, which gives us the confidence to further complete our method. In view of wrong segmentation still existing, it is not realistic to let clinicians evaluate every automatic segmentation result. Even though specialists find some wrong connections in PDA, will only affect imaging analysis to some degree. However, wrong connections happened in complex CHD like TOF, TGA, and SV, are very different from the ground truths. As our goal is to achieve fully automatic segmentation and reconstruction of the CHD model that can guide surgery, the lack of clinical-level criterion will seriously affect the future development of our method. Although our clinical evaluation used in this paper is not excellent, we will try to solve those problems in future work.

## Conclusion

In this paper, we proposed a whole heart and great vessel segmentation framework for CT images in CHD. We first used deep learning to segment the four chambers and myocardium followed by the blood pool, where variations were usually small. We then extracted the connection information and applied graph matching to determine the categories of all the vessels. We collected a CHD dataset in CT with 68 3D images, and the ground truth has seven categories: LV, RV, LA, RA, myocardium, Ao, and PA. 14 types of CHD are included in this dataset which is made publicly available. Compared with the state-of-the-art method for whole heart and great vessel segmentation in normal anatomy, our method has achieved 12% improvement in Dice score on average, and well performance when evaluated by clinical evaluation. We hope these results may pave the way for the clinical use of our method in the incoming future. Due to the limited GPU memory, currently, we used a 3D U-net to process a down-sampled CT image, which cannot segment the related structures precisely. In the future, with more advanced GPUs, we may try to process CT images with much larger dimensions. A fusion (or an ensemble) network should be further adapted to take the outputs of the 2D U-net and the 3D U-net and output the final segmentation. In addition, a two-stage training strategy should be applied in which the 2D U-net and the 3D U-net are firstly trained, and then all three networks are fine-tuned together. In this way, the segmentation accuracy can be further improved potentially.

## Data Availability

The datasets generated during and/or analyzed during the current study are not publicly available due to patient privacy protection but are available from the corresponding author upon reasonable request.

## Code availability

Correspondence and requests for materials and code could be addressed to Dr. Xiaowei Xu upon reasonable request.
